# Loss of p24 function in *Drosophila melanogaster *causes a stress response and increased levels of NF-κB-regulated gene products

**DOI:** 10.1186/1471-2164-9-212

**Published:** 2008-05-08

**Authors:** Kara A Boltz, Ginger E Carney

**Affiliations:** 1 Department of Biology, Texas A&M University, College Station, TX, USA

## Abstract

**Background:**

Secretory and transmembrane proteins traverse the endoplasmic reticulum (ER) and Golgi compartments for final maturation prior to reaching their functional destinations. Members of the p24 protein family, which are transmembrane constituents of ER and Golgi-derived transport vesicles, function in trafficking some secretory proteins in yeast and higher eukaryotes. Yeast p24 mutants have minor secretory defects and induce an ER stress response that likely results from accumulation of proteins in the ER due to disrupted trafficking. We tested the hypothesis that loss of *Drosophila melanogaster *p24 protein function causes a transcriptional response characteristic of ER stress activation.

**Results:**

We performed genome-wide profiling experiments on tissues from *Drosophila *females with a mutation in the *p24 *gene *logjam *(*loj*) and identified changes in message levels for 641 genes. We found that *loj *mutants have expression profiles consistent with activation of stress responses. Of particular note is our observation that approximately 20% of the loci up regulated in *loj *mutants are *Drosophila *immune-regulated genes (DIRGs), many of which are transcriptional targets of NF-κB or JNK signaling pathways.

**Conclusion:**

The *loj *mutant expression profiling data support the hypothesis that loss of p24 function causes a stress response. Genes involved in ameliorating stress, such as those encoding products involved in proteolysis, metabolism and protein folding, are differentially expressed in *loj *mutants compared to controls. Nearly 20% of the genes with increased message levels in the *loj *mutant are transcriptional targets of *Drosophila *NF-κB proteins. Activation of NF-κB transcription factors is the hallmark of an ER stress response called the ER overload response. Therefore, our data are consistent with the hypothesis that *Drosophila *p24 mutations induce stress, possibly via activation of ER stress response pathways. Because of the molecular and genetic tools available for *Drosophila*, the fly will be a useful system for investigating the tissue-specific functions of p24 proteins and for determining the how disrupting these molecules causes stress responses in vivo.

## Background

Proper functioning of the ER is critical for cell survival and function. Perturbations in protein folding in the ER or in trafficking of secretory proteins are associated with a large number of human maladies, including diabetes and neurodegenerative diseases, and can lead to chronic inflammation and cancer [Reviewed in [1-5]]. The ER is the first cellular compartment in which secretory and membrane proteins undergo post-translational modification as they progress through the cellular membrane systems to their final functional location. When ER homeostasis is disrupted or trafficking is blocked, cells attempt to correct the problem via induction of stress response pathways such as the unfolded protein response (UPR) and the ER overload response (EOR), phylogenetically conserved mechanisms for dealing with cellular assaults [Reviewed in [4,6-12]].

The UPR and EOR signaling programs can be activated by the presence of unusually large amounts of proteins within the ER that are not trafficked to the Golgi. For instance, the UPR is induced when mutant proteins cannot be properly folded and accumulate within the ER lumen. The EOR, which is characterized by activation of the Rel transcription factor NF-κB and its downstream signaling pathways, is stimulated by events such as viral infection that produce overwhelming amounts of non-mutant proteins [Reviewed in [13]].

The UPR attenuates translation to decrease overall protein load in the ER and activates transcription of genes encoding chaperones, oxidoreductases, and other proteins necessary for folding and post-translational modification. Genes involved in protein metabolism are up regulated as well. The activated UPR also targets improperly folded proteins to the ER-associated degradation (ERAD) pathway and up regulates genes necessary for protein metabolism. Cells unable to overcome ER stress may ultimately die by apoptosis [Reviewed in [4,6-10]].

Three cellular signal transduction pathways mediate downstream UPR or EOR events [Reviewed in [4,6-10]]. In mammals PERK, Atf6 and Ire-1 are ER transmembrane proteins, with N-termini in the ER lumen and C-termini in the cytoplasm, which serve as sensors for changes within the ER, including alterations in calcium levels, redox environment or levels of nascent proteins. Translational attenuation, which decreases the overall amount of nascent protein requiring ER modification and folding, is mediated by PERK kinase activation by autophosphorylation. Subsequent PERK inactivation of the eIF2α translation initiation factor via phosphorylation is responsible for the resulting translational attenuation.

Upon UPR induction, the Ire-1 endonuclease is also phosphorylated, allowing cleavage of the *Xbp1 *mRNA to produce a functional bZIP transcription factor that positively regulates expression of downstream genes such as ER chaperones. Ire-1 also targets a subset of mRNAs encoding membrane and secreted proteins for degradation [14]. The third UPR signal, Atf6 is a transcription factor that is released from the ER during cellular stress, moves to the Golgi for final modification, and then activates transcription of downstream genes including chaperones and *Xbp1*. There are *Drosophila *counterparts to PERK (PEK in *Drosophila*), Atf6, Ire-1 and Xbp1 [[15-17], Reviewed in [11]], although the genes and their targets are largely unstudied in this model system.

One or more of the signaling cascades involved in the UPR also activate the EOR and NF-κB. This NF-κB activation occurs post-translationally, possibly as a result of PERK-mediated translational attenuation of the NF-κB inhibitory partner I-κB [18,19]. Other data suggest a model in which NF-κB is a downstream target of Ire-1 through an interaction between Ire-1 and the E3 ubiquitin ligase Traf2 that leads to NF-κB activation [20]. Yeast do not have NF-κB homologs and lack two of the UPR signaling pathways found in multi-cellular organisms; all known UPR signaling in yeast is regulated by Ire-1.

It is not clear that activation of the UPR and EOR are entirely separable events since both responses require components of the same ER stress-activated signaling cascades [21]. Several chemical agents that induce the UPR also trigger the EOR, although some inducers may be pathway specific [Reviewed in [13]]. One possibility is that the EOR is a later response to stresses that cannot be overcome by the initial UPR activation [21]. Early UPR activation may induce "adaptation genes", and, if the stress cannot be overcome, the "alarm genes" characteristic of the EOR may be induced in a final effort to save cells before they initiate apoptosis [21].

Members of the p24 protein family are constituents of ER and Golgi-derived vesicles and are hypothesized to function in a variety of ways in intracellular protein trafficking [Reviewed in [22]]. Since loss of p24 function reduces trafficking of specific proteins [23-25] and induces the UPR in yeast [26], we reasoned that loss of p24 expression in *Drosophila *should similarly reduce protein trafficking and induce an ER stress response. Because we expected trafficking of otherwise normal proteins to be affected in p24 mutants, we further predicted that the *Drosophila *stress response would differ from that in yeast by more closely resembling the EOR rather than the UPR and that the response would be activated by the PEK or Ire-1 pathways. Our genome-wide expression profiling and genetic interaction studies are consistent with the hypotheses that loss of p24 function leads to activation of stress responses in metazoans and that both the PEK and Ire-1 pathways may be involved in these responses through activation of the NF-κB proteins Dif and Rel.

## Results

Since deletion of a yeast p24 protein induces an ER stress response [26], we predicted that loss of a *Drosophila *p24 protein, Loj, would elicit a similar response in flies. Because flies contain all three stress response pathways (PEK, Atf6, Ire-1) while yeast only have the Ire-1-mediated response, we expected that loss of trafficking due to a p24 mutation might induce multiple ER stress-activated signaling pathways. Since one characteristic of ER stress responses is transcriptional activation of numerous gene products to ameliorate the stress, we utilized a genomic profiling approach to determine the genes with altered expression in *loj*^00898^/*loj*^04026 ^mutants (henceforth referred to as *loj *mutants) compared to controls. We showed previously that this allelic combination is strongly hypomorphic and probably genetically null [27].

*loj *transcripts and proteins are highly expressed in the follicle cells of developing eggs and in the central nervous system (CNS) [27,28] as well as in other tissues such as the gut and fat body (K.A. Boltz, S. Grady, and G.E. Carney, unpublished results). For our analyses, female abdomens containing the ovaries, genital tract, fat, and gut tissue were dissected away from the remainder of the body (head/thorax), which contains the entire CNS as well as the fat surrounding the head. Mutant and control samples from either the abdomen or head/thorax were used to generate labeled samples for microarray analyses using Affymetrix *Drosophila *Genome 2.0 arrays. We identified a similar number of expression changes in each of the two samples using a 1.5-fold cut-off (p < 0.001 for all data extraction methods for each sample). In the abdominal preparations 366 genes showed transcriptional changes, and 372 genes were identified in the head/thorax samples. Ninety-seven of the genes are common to both tissue preparations (Table 1).

**Table 1:** Total number of genes with altered expression levels in *loj *mutant tissues

	Up regulated	Down regulated	Total changes
Abdomen only	205	64	269
Head/thorax only	164	111	275
Common to both tissues	77	20	97

Total	446	195	641

Of the 641 genes showing transcriptional changes in one or both tissues, approximately one fifth (131 genes) are up regulated by various pathogens or septic injury (Table 2); many are targets of the Toll or Imd signaling cascades [29-37]. The Toll pathway in *Drosophila *regulates immune response to pathogens and septic injury by activating the NF-κB transcription factors Dorsal (Dl) and Dif [38-41], while the Imd pathway branches downstream of TAK1 to activate Jun kinase (JNK) and Relish (Rel) signaling in response to pathogens or injury [31]. Rel is the third NF-κB transcription factor in *Drosophila *that controls humoral immune responses [42]. Since activation of NF-κB is the hallmark of the EOR, our data showing increased levels of numerous immune responsive and NF-κB target genes suggest that a stress response that includes the EOR is activated in *loj *mutant females.

**Table 2:** Total DIRGs with altered expression levels in *loj *mutant tissues

	Up regulated	Down regulated	Totals
Abdomen only DIRGs	44	3	47
Head/thorax only DIRGs	44	4	48
DIRGs common to both tissues	36	0	36

Total	124	7	131

### Expression changes common to both tissues

While the majority of the genes identified in our analyses were specific to either the abdomen or the head/thorax samples, numerous changes were common to both tissue preparations. Twenty genes had decreased levels (including *loj*, see Additional file 1) in both mutant tissue samples, while 77 genes showed increased expression in *loj *mutants compared to controls (see Additional files 1 and 2). Seventeen of these 97 genes were found previously to be transcriptionally responsive to the ER stress inducing agent tunicamycin [43]. Fourteen of our genes show a similar response to tunicamycin-induced changes, while 3 genes show the opposite pattern (see Additional files 1 and 2). *Tequila*, which is down regulated in *loj *mutants (see Additional file 1), is also repressed in tissue culture cells in which the UPR has been induced by dithiothreitol treatment; this decrease is dependent upon Ire-1 but not Xbp1 [14].

Of the genes identified in both tissue data sets, 36 are known or likely *Drosophila *immune-regulated genes (DIRGs), the majority of which are targets of Toll or Imd signaling (see Additional file 2). *GADD45β *is an ER stress response gene that is regulated by NF-κB and exerts an anti-apoptotic effect by down regulating JNK signaling [44]. Also up regulated in this common data set are genes encoding small immune peptides such as Cecropins and Attacins as well as the gram-positive bacteria peptidoglycan receptor *PGRP-SA *(see Additional file 2). The NF-κB gene *Rel *also has increased transcript levels in the *loj *mutant tissues.

To validate our microarray data, we used qPCR to test a subset of genes with altered expression profiles in both tissue samples. Each tissue was assayed independently and showed the expected directional change (see Methods and Additional files 1 and 2). For most of the genes tested, the gene expression levels are significantly different between *loj *mutants and controls in both tissues (p < 0.05). In only one instance (*GstD5*) was the difference non significant for either tissue.

### Mutations in the p24 gene eclair also have elevated levels of immune-regulated products

We anticipated that increased expression of immune responsive and NF-κB target genes would be a general response to *p24 *gene mutations in multi-cellular animals. In *Drosophila*, mutations in two other *p24 *genes, *eclair *(*eca*) and *baiser *(*bai*), cause oviposition defects similar to those observed for *loj *mutants [27,45]. Both *eca *and *bai *are co-expressed with *loj *in ovarian follicle cells and are expressed in the CNS [28].

We generated viable *eca *mutant females and tested them for increased expression of four of the immune regulated genes that are up regulated in *loj *mutants (See Methods). Expression of *CG6687*, *Tsp42Ed *and *Frost *is significantly increased in *eca *mutants relative to controls, while *Socs36E *is not significantly increased in *eca *mutants (data not shown). These results indicate that activation of NF-κB and immune-regulated genes may be a general response to *p24 *loss-of-function mutations.

### Expression changes in the abdomen

The egg tissue of the *Drosophila *abdomen expresses Loj throughout all stages of development, with particularly strong expression observed at stage 10 in the somatically-derived follicle cell layer [27,28], whose primary function is secreting factors needed for eggshell formation. Low-level Loj expression is also observed in the nurse cells [27,28] that produce mRNAs and other factors necessary for embryonic development. Our recent analysis indicates that the Loj protein is highly expressed in other adult abdominal tissues as well, including the fat body and the gut (K.A. Boltz, S. Grady, and G.E. Carney, unpublished results).

We identified a set of 366 genes with altered expression patterns in *loj *mutant abdominal tissue. Of these loci, 269 were noted only in the abdomen while the remaining 97 were also detected in the head/thorax samples (Table 1; see Additional files 3 and 4). Forty-seven of the 269 abdomen-specific genes were previously determined to be immune responsive. Most are transcriptional targets of Toll or Imd signaling while others are components of JNK signaling pathways (see Additional file 3). General immune-responsive genes are overrepresented in this data set (p = 4.915 × 10^-17^, Fisher's Exact Test) as are components of JNK signaling (p = 9.192 × 10^-5^, Fisher's Exact Test).

The abdominal tissue also differentially expressed 222 genes with no known function in immunity (see Additional file 4). Most of the published genome profiling experiments that identified immune response genes used an earlier version of the *Drosophila *genome array [29-32,35] that lacked many of the transcriptional units on the *Drosophila *Genome 2.0 chips used in this study. Therefore, some of the genes in Additional file 4 likely have previously unrecognized roles in the immune response.

Genes in the up-regulated class have functions consistent with involvement in a UPR response (see Additional file 4). *CG14207 *encodes a small Hsp20-like chaperone. *CG33486 *encodes an asparagine synthetase, a protein previously implicated in the mammalian ER stress response [46]. The predicted function of *CG4415 *is unfolded protein binding, suggesting it functions in the UPR as well. Many other up-regulated genes encode proteins with predicted functions such as proteolysis and peptidolysis, metabolism and oxidoreductase activity.

### Expression changes in the head/thorax

A subset of cells in the CNS also has high-level Loj expression, and CNS expression of *loj *in *loj *mutants rescues egg laying and fertility [28]. We separated the *loj *CNS effects from those occurring in developing eggs and gut by dissecting the abdomen (containing the ovaries and gut but not CNS tissue) away from the remainder of the fly.

We identified a set of 275 genes differentially regulated in these samples compared to those from abdomens (Table 1; see Additional files 5 and 6). Many up-regulated genes function in processes such as lipid and protein metabolism. Again, approximately 20% of the genes have been implicated in immune signaling (Table 2; see Additional file 5). Immune responsive as well as JNK pathway components are also overrepresented in the head/thorax preparations (Immune gene p = 1.594 × 10^-19^; JNK p = 3.110 × 10^-3^; Fisher's Exact Test).

### loj interacts genetically with members of the NF-κB genetic signaling pathways

The Toll and Imd pathways activate different, but sometimes overlapping, immune-responsive genes depending upon the pathogen [32,47]. In adult flies, the NF-κB protein Dif is the Toll signaling effecter molecule in response to immune challenge, while the adult function of Dl is not clear. Rel functions downstream of Imd signaling to activate DIRGs [Reviewed in [48]]. The fact that we observed increased message levels of signaling molecules that lie downstream of each pathway begs the question as to whether the three Drosophila NF-κB proteins are activated in the *loj *mutant. Therefore, we tested for genetic interactions between *loj *and the three *Drosophila *NF-κB genes to identify which may be involved in the stress response.

Since *loj *is expressed throughout development [27], we anticipated that activation of the ER stress response in *loj *mutants would be important for survival to adult eclosion from the pupal case. If NF-κB proteins are needed for stress response activation to survive pupation, we expected that reducing NF-κB levels in the *loj *mutant would affect adult survival. When we assayed eclosion rates in double mutant combinations of *loj *with *Dif*, *dl *or *Rel*, we found that fewer than the expected number of *Dif; loj *or *loj, Rel *mutant offspring emerged from the pupal case (Table 3). These results suggest that the Dif and Rel proteins are needed in *loj *mutants to ameliorate the stress response so that animals can survive development.

**Table 3:** *loj *genetically interacts with *Drosophila *NF-κB genes *Dif *and *Rel*

**Genotype**	**# eclosed**	**% expected eclosing**
*Dif^2^/+; loj^00898^/+*	369	100%
*Dif^2^; loj^00898^/+*	192	52.0%
*Dif^2^/+; loj^00898^*	122	33.1%
*Dif^2^; loj^00898^*	18	4.9%
		
*Dif^2^/+; loj/+*	94	100%
*Dif^2^; loj/+*	47	50.0%
*Dif^2^/+; loj^00898^/loj^04026^*	55	58.5%
*Dif^2^; loj^00898^/loj^04026^*	14	14.8%
		
*dl^1 ^cn^1 ^sca^1^/+; loj^00898^/+*	94	100%
*dl^1 ^cn^1 ^sca^1^; loj^00898^/+*	10	10.6%
*dl^1 ^cn^1 ^sca^1^/+; loj^00898^*	13	13.8%
*dl^1 ^cn^1 ^sca^1^; loj^00898^*	11	11.7%
		
*loj^00898^*, *Rel^E20^/+*	315	100%
*loj^00898^*, *Rel^E20^*	6	1.9%
		
*loj^00898^*, *Rel^E20 ^*or *loj*^00898^*/+*	135	100%
*loj^00898^*, *Rel^E20^/loj^00898^*	71	100.5%
		
*loj^00898^*, *Rel^E20 ^*or *Rel^E20^/+*	57	100%
*loj^00898^*, *Rel^E20^*/*Rel^E20^*	20	70.0%
		
*imd^EY08573^/+; loj^00898^/+*	111	100%
*imd^EY08573^; loj^00898^/+*	60	54.0%
*imd^EY08573^/+; loj^00898^*	17	15.3%
*imd^EY08573^; loj^00898^*	17	15.3%

For tests with *Dif, dl*, and *imd *each of the four expected progeny classes should be represented at equal frequency (25% each). Only two types of progeny are produced in the *loj*^00898^, *Rel*^*E*20^*/TM3, Sb *cross, so each class should account for 50% of the offspring. Homozygous *loj*^00898^, *loj*^04026^, *Dif*^2 ^or *dl*^1 ^mutations independently affect adult viability, while *Rel*^*E*20 ^and *imd*^*EY*08573 ^stocks are maintained as homozygotes. Adult eclosion is dramatically reduced when *Dif*^2 ^or *Rel*^*E*20 ^(but not *dl*^1 ^or *imd*^*EY*08573^) homozygous mutations are introduced into the *loj*^00898 ^homozygous mutant background. Similar results were obtained for 5 independent *loj*^00898^, *Rel*^*E*20 ^recombinant strains and different genetic backgrounds containing *imd*^*EY*08573^. Large numbers of *loj*^00898^, *Rel*^*E*20^*/Rel*^*E*20 ^and *loj*^00898^, *Rel*^*E*20^*/loj*^00898 ^(or *loj*^04026^) survive to adulthood (70–100% of expected), indicating that the decreased eclosion of *loj*^00898^, *Rel*^*E*20 ^animals is largely due to effects from mutations in both genes. Although homozygous mutations in *loj *or *dl *decrease adult viability, we did not observe additional, large effects when both genes are mutated in 2 different genetic backgrounds.

## Discussion

When the ER has an unusually high burden of protein products due to viral infection, mutation, or other assaults, mechanisms are activated to reduce the ER protein load. Our results that stress response genes are activated in p24 mutants suggest that loss of p24 protein function causes stress, possibly by disrupting ER homeostasis. Furthermore, we show that a large proportion of the genes with altered transcriptional profiles in a *loj *p24 mutant genetic background are known or suspected targets of immune signaling pathways regulated by the *Drosophila *NF-κB proteins Dif and Relish; function of each of these proteins is needed for *loj *mutants to survive to adulthood. Activation of NF-κB is the signature event in the EOR response to increased ER protein load.

### The loj p24 mutant activates stress response genes

We discovered transcript-level changes in genes encoding factors involved in protein metabolism and folding that are consistent with induction of the UPR. However, we did not identify all of the genes found in a previous study on tunicamycin-induced ER stress in *Drosophila *[43], which identified approximately 600 transcriptional changes in untreated compared to tunicamycin-treated flies using a 1.5-fold change threshold. Tunicamycin activates the UPR as well as the EOR [13].

In yeast, loss of p24 function results in increased splicing of the Ire-1 target *Xbp1 *[26]. We did not find evidence for increased transcription of *Xbp1 *in our p24 mutant microarray experiment, which is consistent with the Girardot *et al*. report (2004) showing that the UPR transcriptional target *Xbp1 *is not up regulated in tunicamycin-treated flies. Furthermore, a second RT-PCR-based assay did not reveal increased splicing of *Xbp1 *in *loj *compared to control animals (data not shown) nor did we observe activation of *Xbp1-EGFP *[16] expression in *loj *mutants. The fact that increased *Xbp1 *transcription and splicing are not detected could be because these responses occur prior to the time point of our assays. Alternatively, some aspects of the tunicamycin and p24 mutant stress responses may be independent of *Xbp1 *signaling. Activated Atf6 increases expression of *Xbp1 *during ER stress [49], but Atf6 signaling has not been implicated in NF-κB activation during the EOR.

Our study identified 641 expression changes, 92 of which were demonstrated previously to be tunicamycin sensitive. Sixty-five transcripts had the same directionality as in Girardot *et al*. (2004) [43], while the remaining 27 show opposite patterns (see Additional files 1, 2, 3, 4, 5, 6). The tunicamycin experiment differed from ours in that the researchers used wild-type *Drosophila *adult male bodies and probed an earlier version of the *Drosophila *genome array with lower transcriptional coverage. Some of the differences in the two studies may be due to sex, genetic background and assayed transcripts. Additionally, the *loj *mutants should be chronically stressed due to loss of p24 function throughout development; the tunicamycin-treated animals experienced an acute stress response since they were assayed 24 hrs after being placed on sugar medium containing tunicamycin [43]. Therefore, we expect that many differences in the two studies are due to differential effects from chronic compared to acute stress responses.

Our most interesting finding is the large number of immune-responsive genes with altered transcriptional profiles in the *loj *p24 mutant. Since the majority of currently published studies on *Drosophila *immune-responsive genes used the Affymetrix version 1 *Drosophila *Genome Arrays (based upon Berkeley Drosophila Genome Project v4.0), they could not identify all immune-regulated loci. Based upon their predicted functions, immune-regulated candidates from our study include *Cht4 *(see Additional file 1), *ImpL2 *and *CG33093 *(see Additional file 4).

### loj interacts genetically with Dif and Rel

Recognizing that increased DIRG message levels could be explained by activation of one or more Drosophila NF-κB proteins, we tested for genetic interactions between *loj *and *Dif*, *dl *or *Rel*. We found that *Dif*^2^*; loj *and *loj*^00898^, *Rel*^*E*20 ^mutant combinations dramatically reduced adult eclosion, while effects of the *dl*^1^*; loj*^00898 ^double mutant combination did not differ from *dl*^1^*; loj*^00898^/+ or *dl*^1^*/+; loj*^00898^. These results indicate that NF-κB and *loj *mutations interact with one another. The results suggest that the *loj *mutant stress response is needed for *loj *mutant animals to survive development and that this response is partially mediated by Dif and Rel. It is possible that the two mutations independently make a "sick" fly worse off. However, *Dif *and *Rel *are not required for viability, and neither *dl *nor *imd *mutations enhance the *loj *mutant phenotype, although both genes affect viability in *loj/+ *animals.

In our study we did not identify expression changes in all of the known immune-regulated genes. If both Dif and Rel are activated in the *loj *mutant, one might expect that all potential NF-κB target genes should be affected. However, NF-κB target sites in immune-responsive promoters are variably responsive to NF-κB and can be modulated by other factors to provide specialized immune responses [50], possibly in a tissue-specific manner. Therefore, the *loj *mutant may not activate all possible NF-κB targets because other cellular factors are needed for a strong response.

### Mechanisms for stress-induced activation of NF-κB

In mammals two potential mechanisms for ER stress-induced NF-κB activation have been described. The first involves PERK-mediated translational inhibition of the NF-κB inhibitor protein IκB [18,19]. In *Drosophila *loss of IκB could only affect Dif signaling since Dif, but not Rel, is maintained in an inactive form through an association with the *Drosophila *IκB protein Cactus [48]. Degradation of Cactus and release of its inhibitory effect on Dif allows downstream transcriptional changes. In contrast, the *Drosophila *NF-κB transcription factor Rel is a bi-partite protein containing inhibitory as well as activating domains. Similarly to the mammalian p105 and p100 NF-κB proteins, Rel activation involves a proteosome-mediated cleavage event that releases the inhibitory domain from the activating domain. Therefore, it is unlikely that PEK-mediated translational attenuation of an inhibitor molecule functions in Rel activation.

The second proposed mechanism for mammalian NF-κB activation due to ER stress is mediated by Ire-1 via an interaction with the TNF-receptor-associated factor 2 (Traf2) [20]. Traf2 is an E3 ubiquitin ligase that activates NF-κB and JNK signaling [[51], Reviewed in [52]]. *Drosophila *encodes a Traf2-like molecule, and there is evidence that Drosophila Traf proteins are involved in Toll, Imd and JNK signaling [53-56]. One possible scenario for NF-κB activation due to ER stress in *Drosophila *is that Ire-1 modulates the Rel-induced ER stress pathways while Pek translational attenuation regulates Dif signaling.

In yeast, loss of p24 protein function causes an ER stress response, one consequence of which is secretion of the heat shock protein and ER stress sensor BiP [26]. Interestingly, there is evidence in mammals that BiP and related heat shock proteins bind to and activate Toll-like receptors [Reviewed in [57-59]]. Therefore, it may be possible to induce NF-κB independently of PEK, Atf6 or Ire-1 if BiP is secreted. In *Drosophila*, the Toll receptor regulates Dif and Dorsal signaling. Even if BiP activation of Toll receptors plays a role in stress-induced NF-κB activation, it cannot account for the observation that *Rel*, which is not regulated by Toll signaling, is induced and interacts genetically with *loj*. Since *imd *regulates *Rel *but does not interact genetically with *loj*, Rel signaling in this p24 mutant context could be mediated by the Ire-1/Traf2 pathway rather than via Imd. Similarly, the Ire-1/Traf2 pathway may modulate the JNK response genes that are up regulated in *loj *mutants (see Additional files 2, 3 and 5). Because Rel activation requires Rel cleavage rather than disruption of an inhibitory protein interaction, it is unlikely that Rel activation is immediately downstream of Pek.

Our analysis did not identify the same set of stress-responsive genes previously shown to require Ire-1 but not other components of the UPR/EOR response [14]. Only one locus, *Tequila*, is down regulated in both experiments. However this difference in affected genes may be due to the stressors or the time periods examined.

There are other possibilities for the observed increases in NF-κB target genes in the *loj *mutant. One is that the observed stress response results from tissue damage occurring in *loj *mutants. Tissue damage could activate NF-κB and stress response genes in the fat body. Another possibility is that increased levels are due to increased message stability rather than activation of NF-κB in the adult. We cannot rule out the possibility that changes in stability of NF-κB target gene transcripts, rather than NF-κB activation, account for the observed expression differences between *loj *and wild-type females.

### Do loj p24 mutants have a trafficking defect?

Since *loj *mutants have increased expression of many genes implicated in ER stress responses and *loj *mutations interact genetically with mutations in two NF-κB genes, it is likely that intracellular trafficking is reduced in *loj *mutants. Genes that encode components of the eggshell (*dec-1*, *Vm32E *and *Cp15*) are down regulated in abdominal tissue (see Additional file 4). It is possible that trafficking of these proteins or is slowed in *loj *mutants and provides feedback inhibition of transcription. Alternatively, these products may be decreased in the *loj *mutant because egg production is slowed and fewer eggs of the stages that express these genes are present in *loj *females.

Another interesting finding is that transcript levels of the octopamine receptor Oamb are also decreased in the *loj *mutant (see Additional file 4). Octopamine signaling is required for egg laying in flies and other insects [60-62], and reduced Oamb protein expression impairs female ovulation [63]. Loss of octopamine signaling via decreased trafficking of the receptor or molecules involved in octopamine or Oamb production could account for the egg-laying defect in *loj *mutants.

In both *loj *mutant tissue samples the *p24-2 *gene (*CG33105*) is up regulated (see Additional file 1). *p24-2 *is a member of the Drosophila p24-alpha subfamily, while *loj *is a member of the p24-gamma subfamily [22,28]. It is possible that *p24-2 *can substitute for some *loj *functions during development or in the adult. We plan to test these hypotheses during future investigations into p24 protein function in flies.

## Conclusion

The genetic profiling data support our assertion that loss of p24 function induces a stress response that includes increasing the levels of NF-κB target gene transcripts, a hallmark of the EOR. Furthermore, the decreased viability of double mutants for *loj *and *Dif *or *Rel *suggests that these NF-kB proteins and their downstream signaling pathways play important roles in ameliorating the stress response.

## Methods

### Drosophila Stocks

*Drosophila melanogaster *stocks and cross progeny were maintained at 25°C on a 12 h light-dark cycle using a standard cornmeal, sugar, agar and yeast culture medium. The *loj*^00898 ^and *loj*^04026 ^alleles were described previously [27]. The *eca*^1 ^allele was provided by S. Bartoszewski [45]. *dl*^1 ^and *Df(3R)GB104*, *red/TM3*, *Sb Ser *(which removes *eca*) were obtained from the Bloomington *Drosophila *Stock Center (stock numbers 3236 and 1937, respectively). The *Dif*^2 ^[41] and *Rel*^*E*20 ^[42] alleles used in this study were provided by B. J. Taylor. A description of each allele is available in Flybase (see Availability and requirements section for URL).

### Microarray Analyses

Females of the genotypes *loj*^00898^/*loj*^04026^, *loj*^00898^/+, and *loj*^04026^/+ were collected as virgins within 1–2 h of the beginning of the 12 h lighted portion of the light-dark cycle, kept in glass vials in groups of 20 or fewer flies, and aspirated singly into new vials on day 3 after collection. On day 4, a single wild-type male was aspirated into a vial containing a single female and observed for mating. Only females that mated within 30 min for 18–30 min were collected for subsequent RNA extractions. Female abdomens were dissected away from the head/thorax 3 hours after the end of the mating period and each of the two tissue types (abdomen or head with thorax) was quick-frozen in Trizol reagent (Invitrogen). Total RNA from abdomen or head/thorax tissue was extracted in Trizol using the manufacturer's protocol. Twelve female tissue samples were collected for each of 6 independent RNA extractions for *loj*^00898^/*loj*^04026^. Our control samples consisted of 6 *loj*^00898^/+ and 6 *loj*^04026^/+ samples combined together for each of 6 independent RNA extractions. Three experimental (*loj*^00898^/*loj*^04026^) and three control RNA samples for each set of tissue were used for array hybridization. Sample labeling and hybridization to *Drosophila *Genome 2.0 Arrays (Affymetrix) was performed at the University of Kentucky Microarray Core Facility using standard Affymetrix protocols for a total of 12 arrays (3 sets of experimental and control samples for each of the two tissues).

Cyber-T was used to perform Bayesian statistical analyses on expression values derived from dChipPM-MM and dChipPM [64] and GCOS (Affymetrix) essentially as described previously [65]. We set stringent parameters and required that the final set of genes with altered expression levels have a significance value of p < 0.001 for all three expression values (dChipPM-MM, dChipPM and GCOS) and show at least a 1.5-fold difference from the control samples. This analysis left us with a data set of 641 genes with expression changes in one or both tissues.

### Real-time PCR (qPCR)

To validate the microarray results, the remaining twelve RNA samples (3 experimental and 3 control preparations for each of the two tissues) were used to prepare independent cDNA samples with the Superscript 1^st ^Strand Synthesis Kit (Invitrogen). cDNA preparations were diluted 1:15, and 1.5 ul was used in each reaction for qPCR using the SYBR Green PCR Mastermix (Applied Biosystems). Reactions were performed in the ABI7700 or ABI7500 Fast Real-time PCR system using the default run parameters. For each plate, a melting curve analysis was performed at the end of each run to test for primer specificity. Additionally, selected reactions were electrophoresed on agarose gels to view PCR products as a second test for presence of the correct product.

Each plate also contained reactions to test for amplification specificity in the presence or absence of template. *rp49 *primers were used for control amplification reactions [27]. cDNAs from abdomen or head/thorax preparations were analyzed separately. We selected 9 up-regulated (*AttC*, *CG6188*, *CG6687*, *CG15829*, *Frost*, *GstD5*, *IM23*, *Socs36E*, *Tsp42Ed*) and 5 down-regulated candidates (*CG8768, CG13793, ninaD, Tequila, UGP*) for this analysis and report the average fold changes from the tissues in Additional files 1 and 2. In every case, samples showed a transcriptional change in the direction expected from microarray analyses and in most instances the differences between *loj *mutants and controls were significantly different (p < 0.05) in both sample preparations. *GstD5 *transcripts were increased in *loj *head/thorax and abdomen samples but the results were not significantly different from controls. Down regulation of *loj *transcripts in *loj *mutant tissue was reported previously [27].

To test *eclair *(*eca*) mutants for increased expression of immune response genes, we performed a similar protocol on cDNA prepared from *eca/Df(3R)GB104 *and control (equal numbers of *eca/+ *and *Df(3R)GB104/+*) females using primers that specifically amplified gene products that are downstream of various stress or immune-activated pathways: *CG6687 *(Toll/Imd), *Tsp42Ed *(Imd/JNK), *Frost *(Toll) and *Socs36E *(JAK/STAT). We were not able to collect *bai *mutant females for analysis due to lethality of *bai *mutations.

### Viability Assays

We determined the number of animals of each of the possible genotypes that eclosed from stock vials for the following strains: (1) *w; Dif*^2^, *cn bw/Cyo, ftz-lacZ; loj*^00898^*/TM3, Sb ftz-lacZ *(701 total animals scored); (2) *loj*^00898^, *Rel*^*E*20^*/TM3, Sb *(321 total animals scored); (3) *dl*^1 ^*cn*^1 ^*sca*^1^*/Cyo; loj*^00898^*/TM2 *(128 total animals scored); (4) *w; imd EY08573/Cyo; loj*^00898^/*TM6b*, *Tb *(205 total animals scored). We also crossed *loj*^00898^, *Rel*^*E*20^*/TM3, Sb *females to either *loj*^00898^*/TM3, Sb *(206 animals scored) or *Rel*^*E*20^*/TM3, Sb *(77 animals scored) males and determined the progeny genotypes. *w; Dif*^2^, *cn bw/Cyo, ftz-lacZ; loj*^00898^*/TM3, Sb ftz-lacZ *was crossed to *w; Dif*^2^, *cn bw/Cyo, ftz-lacZ; loj*^04026^*/TM3, Sb ftz-lacZ *(210 animals scored) to assay interactions between *Dif*^2^and *loj *transheterozygous mutants. A similar cross with *dl*^1 ^in the *loj*^00898^or *loj*^04026 ^background did not produce any *dl*^1^homozygous progeny (data not shown). Stocks were maintained in non-crowded conditions and the number of animals of each genotype that eclosed was determined on multiple days over a multi-week period. Vials were cleared the day before adult offspring were counted.

For strains containing *Dif *or *dl *alleles, each of the 4 possible genotypes from the stock cross is predicted to equally represented due to Mendelian segregation. Since *loj *and *Rel *are both on the 3^rd ^chromosome, there are only two possible genotypes from the stock cross from *loj*^00898^, *Rel*^*E*20^*/TM3, Sb*. The percentage of expected animals eclosing was calculated by dividing the total number of adult animals of a given genotype by the total number of animals of the stock genotype.

From the crosses of *loj*^00898^, *Rel*^*E*20^*/TM3, Sb *to either *loj*^00898^*/TM3, Sb *or *Rel*^*E*20^*/TM3, Sb *there are three classes of expected progeny (*e.g*., (1) *loj*^00898^, *Rel*^*E*20^*/TM3, Sb *(2) *loj*^00898^*/TM3, Sb *and (3) *loj*^00898^, *Rel*^*E*20^/*loj*^00898^) that should each account for one-third of the total number of offspring. We are not able to distinguish the two classes of progeny that contain the *TM3, Sb *chromosome. Therefore, we calculate the percentage of expected *loj*^00898^, *Rel*^*E*20^/*loj*^00898 ^progeny by dividing the total number of *loj*^00898^, *Rel*^*E*20^/*loj*^00898 ^progeny by one-half of the total number of *TM3, Sb *progeny.

## Availability and requirements

Flybase: 

## Authors' contributions

KAB designed and executed the experiments and participated in manuscript preparation. GEC conceived, designed and executed the experiments, participated in the microarray data analysis and wrote the manuscript.

## Supplementary Material

Additional file 1 Gene expression changes common to both tissue preparations. Selected genes were validated by qPCR on independently obtained tissue preparations. * genes identified previously as down regulated in response to a tunicamycin-induced ER overload response (Girardot et al. 2004). ** genes up regulated by a tunicamycin-induced ER overload response (Girardot et al. 2004). (*) indicates an opposite response in *loj *mutants compared to tunicamycin-treated animals. *** greater than 300-fold reduction in transcript levels. Click here for file

Additional file 2 DIRGs common to both tissue preparations. Immune responsive genes are overrepresented in this data set (p = 9.035 × 10^-26^, Fisher's Exact Test) as are components of JNK signaling pathways (p = 4.656 × 10^-4^, Fisher's Exact Test). Click here for file

Additional file 3 Abdomen only DIRGs Click here for file

Additional file 4 Abdomen only changes Click here for file

Additional file 5 Head/thorax only DIRGs Click here for file

Additional file 6 Head/thorax only changes Click here for file
